# Sudden Cardiac Death (SCD) – risk stratification and prediction with molecular biomarkers

**DOI:** 10.1186/s12929-019-0535-8

**Published:** 2019-05-22

**Authors:** Junaida Osman, Shing Cheng Tan, Pey Yee Lee, Teck Yew Low, Rahman Jamal

**Affiliations:** UKM Medical Molecular Biology Institute (UMBI), Universiti Kebangsaan Malaysia, Kuala Lumpur, Malaysia

**Keywords:** sudden cardiac death (SCD), coronary artery disease (CAD), heart failure (HF), coronary heart disease (CHD), cardiovascular disease (CVD), biomarker

## Abstract

Sudden cardiac death (SCD) is a sudden, unexpected death that is caused by the loss of heart function. While SCD affects many patients suffering from coronary artery diseases (CAD) and heart failure (HF), a considerable number of SCD events occur in asymptomatic individuals. Certain risk factors for SCD have been identified and incorporated in different clinical scores, however, risk stratification using such algorithms is only useful for health management rather than for early detection and prediction of future SCD events in high-risk individuals. In this review, we discuss different molecular biomarkers that are used for early detection of SCD. This includes genetic biomarkers, where the majority of them are genomic variants for genes that encode for ion channels. Meanwhile, protein biomarkers often denote proteins that play roles in pathophysiological processes that lead to CAD and HF, notably (i) atherosclerosis that involves oxidative stress and inflammation, as well as (ii) cardiac tissue damage that involves neurohormonal and hemodynamic regulation and myocardial stress. Finally, we outline existing challenges and future directions including the use of OMICS strategy for biomarker discovery and the multimarker panels.

## Background

The heart serves as a biological pump that circulates blood throughout our bodies and thus supplying us with oxygen and nutrients. Within the heart, a heartbeat is first initiated by the sinoatrial (SA) node that releases electrical stimuli. These stimuli traverse the atrioventricular (AV) nodes, the bundle of His, subsequently into the bundle branches and Purkinje fibres, causing the contractions of heart cells called cardiomyocytes. However, these electrical stimuli can sometimes become disorganized, due to ventricular tachycardia or ventricular fibrillation [1]. Irregular cardiac activities restrict blood supply to the brain, causing rapid death of brain cells and leading to sudden cardiac death (SCD) [2, 3]. Globally, SCD accounts for 4–5 million deaths per year [4], and is strongly linked to coronary artery diseases (CAD), especially myocardial infarction (MI) [5]. Other causes for SCD include cardiomyopathies and inherited channelopathies [6].

### Prevention and treatment of SCD

To prevent SCD, implantable cardioverter defibrillator (ICD) is used prophylactically in individuals with existing conditions of cardiomyopathy and inherited arrhythmias. Upon detecting an abnormal heart rhythm, ICD delivers an electric shock to restore normal heartbeats. However, the survival benefits of the ICDs are limited as only 20–30% of patients with ICD receive appropriate therapy [7]. On the other hand, patients with history of MI are recommended to consume beta-blockers, which reduces recurrent MI and angina, but not mortality [8]. Targeting resistant hyper-triglyceridemia is another option. Current European and US guidelines target low-density lipoprotein cholesterol (LDL-C) levels as the primary approach for treatment [9]. However, it was shown that the risk of cardiovascular disease (CVD) increase with excess levels of triglycerides (TG), even in patients with optimally managed LDL-C levels [10].

### Risk factors for SCD

**Fig. 1:**
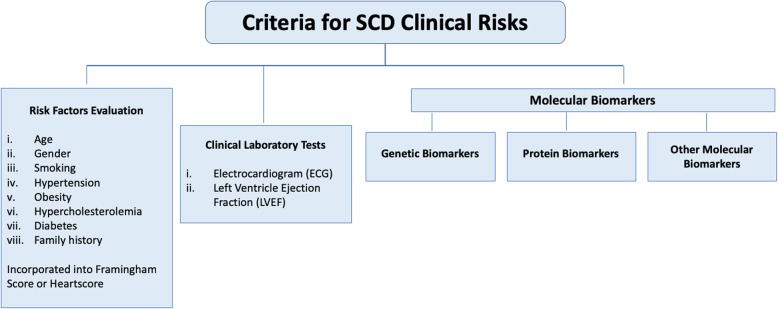
Methods to evaluate and diagnose SCD clinical risks. SCD risks can be evaluated using Framingham risk score or Heartscore, that stratify SCD risks according to the listed criteria. More commonly, diagnosis is performed in the clinics using tests that can detect cardiac symptoms such as abnormal heart rates, electrocardiogram (ECG), or Left Ventricle Ejection Fraction (LVEF). With the advent in molecular medicine, clinical tests are moving towards molecular biomarkers. Genetic biomarkers are effective for screening high-penetrance genome variants that predispose asymptomatic individuals to SCD, for example genes that encode ion channels. On the other hand, protein biomarkers for SCD often depict pathophysiology for coronary artery diseases (CAD) or heart failure (HF). These protein biomarkers are often involved in oxidative stress, inflammation, neurohormonal regulation, hemodynamic properties and myocardial stress. Besides, molecular biomarkers also encompass other biomolecules such as fatty acids and other metabolites

Stratification of clinical risks, including that of SCD, is an important step in effective health management (Fig. 1). Since CAD and heart failure (HF) underly a significant majority of SCD incidence, risk factors for CAD and HF are accepted as predictors for SCD-related deaths and all-cause mortality [11]. In fact, these risk factors, including (i) increased age, (ii) male gender, (iii) cigarette exposure, (iv) hypertension, (v) obesity, (vi) hypercholesterolemia, (vii) diabetes mellitus and (viii) family history have been incorporated into the US-based Framingham Risk Score and Europe-based HeartScore for estimating cardiovascular risks [12, 13]. Apart from those mentioned, other SCD-related risk factors that can be evaluated in clinical laboratories include (i) left ventricle (LV) dysfunction, (ii) history of heart failure (HF), (iii) left ventricular hypertrophy, (iv) poor heart functional status, (v) elevated heart rate and (vi) abnormal electrocardiogram (ECG). Among these, left ventricular ejection fraction (LVEF) measures the blood volume pumped out of the left ventricle using echocardiogram, nuclear magnetic imaging (MRI) or nuclear medicine scan [14]. LVEF classifies HF into (i) reduced (LVEF < 40%), (ii) preserved (LVEF > 50%) and (iii) intermediate (LVEF ~ 40–49%) categories [15]. Meanwhile, ECG measures the rate and rhythm of heartbeats, the size and position of the heart chambers, or detect any injuries to the heart muscle or conduction system. Abnormal ECGs, such as prolonged QT interval, Tpeak–Tend interval and ﻿T-wave alternans have been proposed as risk markers [16, 17]. Nevertheless, besides the lack of consistent association between QT interval prolongation and total or cardiovascular mortality in population-based studies [18], these markers also preclude high-risk individuals without CAD symptoms [7].

### Detecting and screening SCD with molecular biomarkers

The flow of genetic information from genes to RNAs, proteins and metabolites together form the molecular layers that interact with the environment to contribute to biological traits including disease phenotypes [19]. Naturally, these biomolecules are appropriate candidates for “biomarkers”. Biomarkers are objective indicators of normal biological processes, pathogenic processes or pharmacological responses [20]. Ideally, a biomarker should be: (i) sensitive, (ii) specific, (iii) cost-effective, (iv) easily obtainable and (v) non-invasive [21]. Importantly, it should also be (vi) quantifiable, correlate well with the severity of disease conditions and (vii) able to offer early detection.

### Genetic biomarkers

Since many SCD cases are heritable, early genetic studies apply the candidate gene approach to identify potentially meaningful genomic variants that are involved in various predisposing cardiac conditions, such as the long QT syndrome, Brugada syndrome, or cardiomyopathies. Genetic markers are effective for screening high-penetrance genome variants that predispose otherwise asymptomatic individuals to SCD. Early genetic studies had identified such variants by applying the candidate gene approach, whereby candidate genes are first selected based on the functions of wild-type gene products or the biochemical pathway involved in diseases. Association studies are then performed to evaluate variation in the sequences of selected genes predicted to be involved in pathogenesis.

One biomarker that was discovered with this approach is *SCN5A*, which encodes the alpha subunit of the voltage-gated sodium channel Na_v_1.5 [22–24]. Na_v_1.5 regulates the influx of sodium ion, and thus the initiation and propagation of action potentials of the heart. Any variations or mutations in *SCN5A* that affect the structure, function or expression of the sodium channel cause a delayed or persistent entry of sodium ions across the cell membrane, leading to arrhythmogenic syndromes and SCD. Among the SCD-related genetic variations that have been identified in the *SCN5A* gene include: (i) rs7626962 (p.Ser1103Tyr), which causes an amino acid substitution in a conserved sequence between domains II and III of Na_v_1.5 [25]; (ii) rs11720524, which has been predicted to disrupt a transcription factor binding site of the gene [22]; and (iii) rs41312391, that modulates the expression of an adjacent gene that is implicated in the regulation of histone deubiquitinating complexes [26].

Potassium channels play a role in the repolarization of the cardiac action potential [27, 28], and anomalies in the rate of cardiac repolarization can lead to SCD [29]. Notably, *KCNH2* which encodes the K_v_11.1 channel that regulates the rapid component of the delayed rectifier potassium current; and *KCNQ1* which encodes the K_v_7.1 channel that regulates the slow delayed rectifier current are important targets. Several *KCNH2* and *KCNQ1* mutations tare present in long QT syndrome and were documented in SCD [30]. These mutations include the rs199472830 (p.Phe29Leu) and rs199472882 (p.Pro297Ser) mutations of *KCNH2*, as well as the rs120074178 (p.Arg190Trp) mutation of *KCNQ1*. Besides, a study in the Finnish population reveals the occurrence of *KCNH2* rs199472918 (p.Leu552Ser) and rs36210422 (p.Arg176Trp) mutations among three probable SCD cases, although statistical analysis suggested a lack of significant association between the mutations and SCD risk [31]. In addition, Albert et al. showed that the rs2283222 variant of *KNCQ1* gene was significantly associated with an increased risk of SCD [32].

Calcium channels are involved in the excitation-contraction coupling (ECC) process. The cardiac ryanodine receptor (RyR2) is a calcium channel that regulates calcium ion release from the sarcoplasmic reticulum. Activation of RyR2 facilitates binding of calcium ions to contractile proteins of the heart muscle, which activates systolic contraction of the cardiac myocytes [33]. To maintain a regular heartbeat, the activity of RyR2 must be tightly-regulated. Abnormal leak of calcium ions through dysregulated RyR2 can cause an altered membrane potential, which in turn introduce irregular contractile and electrical activity, resulting in cardiac arrhythmia and possibly, SCD [33]. A prominent *RYR2* mutation that has been implicated in SCD is rs3766871 (p.Gly1886Ser), which is present in high prevalence in a molecular autopsy study involving 173 SCD cases, whereby rs3766871 has been demonstrated to result in an increased calcium ion oscillation in the cell and has been postulated to cause diastolic calcium ion leak [23]. As such, it is not surprising that the rs3766871 variant was found to be associated with an almost 2-fold increased risk of SCD.

Mutations in other cardiac-related genes have also been implicated in SCD. These include: *MYBPC3*, which encodes cardiac myosin binding protein C [34]; *ACE*, which encodes angiotensin converting enzyme [35]; *PKP2*, which encodes plakophilin 2 [31]; *DSP*, which encodes desmoplakin [36]. Many of these mutations are rare in the general population, but can contribute to SCD risk in a highly penetrant manner. A collection of genetic biomarkers together with the strength of evidence to indicate their correlation with SCD is shown in Table 1.

**Table 1:** List of genetic biomarkers associated with SCD

Gene	Putative gene function	Association with SCD	SNP/mutation	Strength of evidence^a^	Ref
*SCN5A*	Encodes α subunit of the cardiac voltage-gated sodium channel (Nav1.5)	Variants were associated with SCD	rs7626962 (p.Ser1103Tyr)	++	[25]
			rs11720524		[22]
			rs41312391		[26]
*KCNH2*	Encodes the K_v_11.1 channel that regulates the rapid component of the delayed rectifier potassium current	Variants were associated with SCD	rs199472830 (p.Phe29Leu)	+	[30]
			rs199472882 (p.Pro297Ser)		[30]
		Variants were associated with probable SCD cases	rs199472918 (p.Leu552Ser)	+	[31]
			rs36210422 (p.Arg176Trp)		[31]
*KCNQ1*	Encodes the K_v_7.1 channel that regulates the slow delayed rectifier current	Variant was associated with SCD	rs120074178 (p.Arg190Trp)	+	[30]
		Variant was associated with an increased risk of SCD	rs2283222	+	[32]
*RYR2*	Encodes calcium channel involved in the regulation of calcium ion release from the sarcoplasmic reticulum	Variant was associated with an increased risk of SCD	rs3766871 (p.Gly1886Ser)	++	[23]
*MYBPC3*	Encodes cardiac myosin binding protein C required for normal cardiac function	Variant was associated with an increased risk of SCD	p.F305Pfs^a^27	+	[34]
*ACE*	Encodes angiotensin converting enzyme that catalyzes the conversion of angiotensin I to angiotensin II and the inactivation of bradykinin via the kallikrein-kininogen system	Variant was associated with an increased risk of SCD	DD genotype or D allele	+	[35]
*PKP2*	Encodes plakophilin 2 which is responsible for linking cadherins to intermediate filaments in the cytoskeleton	Variants were associated with arrhythmia disorder and risk of SCD	Q59L	+	[31]
			Q62K		
			N613K		
*DSP*	Encodes desmoplakin that functions to maintain structure integrity	Variants were associated with sudden unexplained nocturnal death syndrome (SUNDS)	rs188516326 (p.Q90R)	+	[36]
			rs116888866 (p.R2639Q)		
			rs200476515 (p.R315C)		
			rs569786610 (p.E1357D)		
			rs185367490 (p.N1234S)		
			rs184154918 (p.R1308Q)		
			rs181378432 (p.T2267S)		
			novel (p.D2579H) (p.I125F) (p.D521A)		

^a^Strength of evidence was rated as “+”: weak, “++”: medium and “+++”: strong based on number of published findings supporting significant correlation of a particular biomarker with SCD, sample size and clinical validity

Nowadays, researchers have gradually switched to genome-wide association studies (GWAS) to validate these variants, and identify novel ones [24, 37]. Nevertheless, different GWAS studies are often inconsistent, which can be due to the heterogeneity in case definitions [38]. To address this inconsistency, a meta-analysis was conducted to discover potential genetic biomarkers of SCD with high statistical power, and found that the *BAZ2B* gene locus was associated with a 1.92-fold increased risk of SCD [37]. Recently, a gene panel targeting 174 expertly-selected genes implicated in inherited cardiac conditions (ICCs) has become commercially available [39]. As ICCs predispose healthy individuals to sudden death, screening SCD cases with this gene panel facilitates high-throughput identification of deleterious variants that underlie SCD. This gene panel has been used in conjunction to another gene panel to perform a molecular autopsy on 302 idiopathic SCD cases (however, only 77 of the 174 genes were analyzed) [40]. After applying robust filtering strategies and stringent criteria for variant classification, it was found that a clinically actionable pathogenic or likely pathogenic variant was present in 13% of the cases. Interestingly, the majority of the pathogenic or likely pathogenic variants resided in *SCN5A*, *KCNH2*, *KCNQ1* and *RYR2* genes as described earlier, which further established these genes as genetic biomarkers of SCD.

### Protein biomarkers

**Fig. 2:**
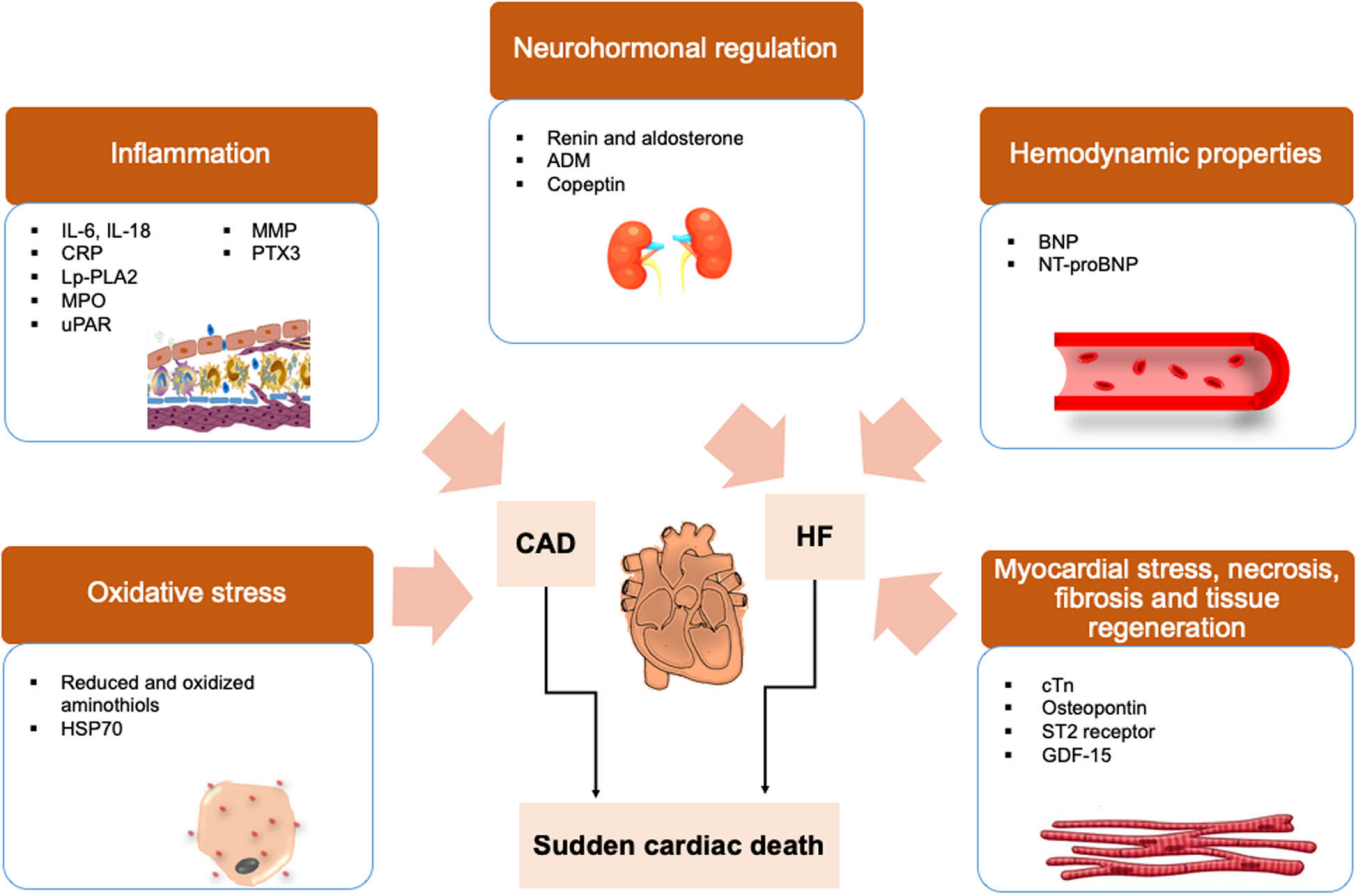
Protein biomarker candidates for assessing risks of SCD. Surrogate biomarkers that reflect the development of oxidative stress and inflammation are associated with CAD (coronary artery disease). While biomarkers that reflect the neurohormonal regulation process, hemodynamic properties and myocardial stress are often associated with HF (heart failure). Both CAD and HF are responsible for sudden cardiac death (SCD)

Proteins are routinely used as analytes in clinical diagnostics. Biofluids, especially plasma and serum are rich and non-invasive sources of circulating proteins that can provide quantifiable readout as biomarkers. Protein biomarkers for cardiac disorders often reflect the underlying pathophysiological processes in CAD or HF, two major causes of SCD (Fig. 2). These pathophysiological processes include (i) oxidative stress, (ii) inflammation that subsequently leads to atherosclerosis, (iii) neurohormonal regulation, (iv) hemodynamic properties, (v) myocardial stress, (vi) necrosis, (vii) fibrosis and (viii) tissue regeneration [41, 42].

### Atherosclerosis and CAD

A major cause of CAD is atherosclerosis, whereby the inside of an artery becomes narrowed due to build-up of plaque. Initially, low-density lipoproteins (LDL) drives atherosclerosis by invading the endothelia of blood vessels, subsequently become trapped in the sub-endothelial space and oxidized by reactive oxygen species (ROS). Oxidized LDLs (oxLDLs) initiate a series of events leading to inflammatory responses [43], build-up of vulnerable plaques, platelet activation, plaque instability, erosion and rupture.

### Oxidative stress biomarkers

As such, oxidative stress represents an initiating event in CAD. It can be assessed by quantifying the levels of plasma aminothiol antioxidants such as cysteine and glutathione and their oxidized counterparts, i.e. cystine and glutathione disulfide [44]. High cystine and low glutathione levels are associated with increased mortality in subjects with CAD [45]. Heat shock proteins (HSPs) are upregulated during oxidative stress [46]. Its levels were demonstrated to be significantly lower in CAD patients, and inversely proportional to the degree of atherosclerosis [47]. However, in a study of 3415 patients with suspected or known CAD undergoing cardiac catheterization, elevated HSP70 levels correlated with increased risk of cardiac death even after adjustment for clinical variables and hsCRP [48].

### Inflammation biomarkers

During atherosclerotic development, following oxidative stress, accumulating oxLDLs recruit monocytes to its residing sub-endothelial space. These transmigrated monocytes subsequently differentiate into macrophages, proliferate locally and ingest oxLDLs, turning into “foam cells” slowly [49]. These macrophages and endothelial cells then release pro-inflammatory cytokines such as interleukin-1 (IL-1), IL-6, IL-8, IL-10 and IL-18 that are involved in T-cell activation [50]. Among these interleukins, IL-6 and IL-18 are established as inflammatory biomarkers that are associated with CAD. In the PRIME study that involved 10,000 asymptomatic European middle-aged men, IL-6 was associated with an increased risk of SCD [51]. Zhao et al. evaluated the relationship of IL-6 with the extent and severity of CAD using coronary computed tomography angiography (CCTA) and detected the association of high IL-6 levels with major adverse cardiac events (MACE) and higher atherosclerotic burden [52]. Cainzos-Achirica et al. explored the prognostic value of IL-6 for the prediction of atherosclerotic cardiovascular disease (ASCVD) events, HF, and other chronic diseases in 6617 participants and concluded that IL-6 is strongly and independently associated with ASCVD events, HF, and all-cause mortality, particularly among statin users [53]. IL-18 is another promising prognostic marker for CAD [54]. Opstad at al. investigated 1001 patients with angiographically verified stable CAD by measuring their circulating IL-18 and IL-12 with ELISA methods [55]. After a 2-year follow-up, 100 cardiovascular endpoints were recorded whereby subjects with simultaneous levels in upper tertiles of both markers were at higher risk of cardiovascular events.

C-reactive protein (CRP) is an acute-phase protein that is secreted by the liver in response to circulating levels of IL-6, IL-1 and TNF-α during the atherosclerotic process [56]. CRP is capable of activating the complement system by binding to phosphocholine molecules on the surface of dead or dying cells [57]. It is also a biomarker for CAD and SCD and can be measured with a high sensitivity CRP (hs-CRP) assay at sub-clinical levels (0.5 to 10 mg/L). The Physicians’ Health Study showed that CRP levels were an independent risk factor for SCD in males after correcting for potential confounders in the general population [58]. In the JUPITER study, randomized statin therapy was given to asymptomatic individuals who manifested elevated levels of hsCRP and LDL, and these individuals experienced 47% reduction in the risk of non-fatal MI, stroke, and cardiovascular death [59]. The BARI-2D trial also discovered a correlation between elevated CRP levels and cardiovascular events [60]. However, there were no observed associations between CRP levels and SCD in the female-based Nurses Health Study and the male-based PRIME study [51, 61].

Lipoprotein-associated phospholipase A2 (Lp-PLA2) is an enzyme that co-travels with circulating LDL, and hydrolyzes oxidized phospholipids in LDL. Lp-PLA2 produces lysophosphatidylcholine and oxidized non-esterified fatty acids, both being bioactive lipid mediators that elicit inflammatory responses [62]. Lp-PLA2 levels were found to independently predict the presence of CAD in the general population, after adjusting for hs-CRP and B-type natriuretic peptide (BNP) [63, 64]. Another oxidaive-stress-related enzyme, myeloperoxidase (MPO), a heme peroxidase, participates in LDL oxidation mediated by radical 1e-oxidation and non-radical 2e-oxidation [65]. Detection, quantification and imaging of MPO mass and activity are useful in cardiac risk stratification [66]. Meanwhile, urokinase-type plasminogen activator receptor (uPAR) is a GPI-anchored membrane protein that, during inflammation, becomes shedded from cell membrane and forms soluble uPAR (suPAR) [67]. The levels of plasma suPAR were shown to correlate with pro-inflammatory markers and even outperform CRP at prognosticating CVD [68, 69]. Another protein, pentraxin-3 (PTX3) is released upon primary inflammatory signals [70] and has been implicated as an inflammatory biomarker for CAD [71]. In two independent clinical trials (CORONA and GISSI-HF) enrolling patients with chronic HF, PTX3 was consistently associated with adverse outcomes [72]. Finally, matrix metalloproteinases (MMP) are implicated in plaque formation and rupture, leading to coronary occlusion [73]. Individuals with acute coronary syndrome and CAD were shown to possess elevated levels of MMP-1, − 2, − 8 and − 9 in their plasma [74, 75].

### Heart failure (HF) and SCD events

Besides CAD, another heart condition that can potentially lead to SCD is heart failure (HF). HF occurs when the heart is unable to pump sufficient blood to supply nutrients and oxygen. In HF, the reduction in cardiac output can be attributed to a cardiac acute injury, a long-standing haemodynamic overload; or genetic variations that disrupt contractile function [76]. The reduction in blood circulation is sensed by peripheral arterial baroreceptors that activate compensatory mechanisms to maintain cardiovascular homeostasis. These compensatory mechanisms include (i) the renin–angiotensin–aldosterone system (RAAS), which maintain cardiac output through increased retention of salt and water, peripheral arterial vasoconstriction and increased contractility; (ii) activation of the adrenergic (sympathetic) nervous system (ANS) to increase heart rate, cardiac contractility and accelerate cardiac relaxation; (iii) secretion of inflammatory mediators and (iv) cardiac repair and remodelling. Certain proteins that are involved in these compensatory mechanisms have been demonstrated to be predictive of SCD.

### Neurohormonal biomarkers

Elevated renin and aldosterone levels were found to be associated with HF and SCD in the LURIC study [77, 78]. Besides, increased aldosterone levels were associated with a higher risk of cardiac arrest in the post–ST-segment elevation MI population [79]. The adrenergic nervous system (ANS) system can also become dysregulated in HF. For example, adrenomedullin (ADM) is a peptide hormone with natriuretic, vasodilatory and hypotensive effects [80] and its concentrations were shown to become elevated in chronic HF [81, 82]. However, since ADM is unstable in vitro, MR-proADM (mid-regional proadrenomedullin), the precursor of ADM is quantified instead in clinical laboratories [83]. In the BACH trial on 1641 patients, MR-proADM identifies patients with high 90-day mortality risks [80, 84]. Another emerging HF biomarker is copeptin. Copeptin is a propeptide fragment of arginine vasopressin (AVP), which mediates vasoconstriction and cardiac hypertrophy. Elevated copeptin is significantly linked to 90-day mortality, readmissions, and emergency department visits, especially in those with hyponatremia [85, 86]. Copeptin was also found to be superior to BNP or N-terminal pro B-type natriuretic peptide (NT-proBNP) as a biomarker for HF; and its increased levels was linked to excess mortality in patients with chronic HF, irrespective of clinical severity [87].

### Hemodynamic biomarkers

During cardiac hemodynamic stress, natriuretic peptides (NP), i.e. BNP or NT-proBNP are secreted. Besides being capable of lowering blood pressure, NPs carry natriuretic, diuretic and kaliuretic properties [88]. NT-proBNP has been reported as an independent risk marker for SCD [61]. This is consistent with another finding that reported an association between higher baseline levels of NT-proBNP and SCD over a 16-year follow-up period [89]. BNP was also independently associated with an elevated risk for SCD in patients with chronic HF in the Vienna Heart Failure Cohort [90] and in survivors of acute MI in the Multiple Risk Factor Analysis Trial [91].

### Myocardial stress biomarkers

Tropomyosin interacts with cardiac *troponin (cTnC, cTnI and cTnT), forming the troponin-tropomyosin complex that* is responsible for cardiac muscle contraction. During myocardial stress, degeneration of the actin and myosin filaments results in the release of cTn into plasma. Therefore, cTnT and cTnI, being unique to the heart, are specific markers for myocardial damage. Both cTn and high sensitivity cTn (hs-cTn) assays have been used as predictors of mortality in both CAD and HF. For instance, elevated levels of hs-cTn have been associated with CAD [92]. In a community-based study, elevated cTn was shown to predict death and first CHD event in 1203 elderly men free from CVD at baseline [93]. Whereas, De Lemos et al. demonstrated that elevated levels of hs-cTn were linked with higher adjusted all-cause mortality in the general population [94]. In the PEACE trial, a graded increase in the cumulative incidence of cardiovascular death in those with higher hs-cTnT levels was observed [95]. On the other hand, as demonstrated by Latini et al., detectable cTnT predicts increased mortality in 4053 patients with chronic HF [96]. Masson et al. also discovered that serial measurements of hs-cTnT concentrations are robust predictors of cardiovascular events in patients with chronic HF [97]. Other noteworthy protein biomarkers that are associated with myocardial stress, necrosis, fibrosis and tissue regeneration are osteopontin (OPN) [98], soluble ST2 receptor [99], and growth differentiator 15 (GDF15) [100]. A list of protein biomarkers and the strength of evidence showing their association with SCD is available in Table 2.

**Table 2:** Summary of protein biomarkers related to various pathophysiological processes that are associated with cardiovascular disease (CVD)

Process	Biomarkers	Association with CVD	Strength of evidence^a^	Ref
Oxidative stress	Reduced (cysteine and glutathione) and oxidized (cystine and glutathione disulphide) aminothiols	High cystine (oxidized) and low glutathione (reduced) levels were associated with higher mortality in patients with CAD	++	[45]
	Heat shock protein 70 (HSP70)	High levels of HSP70 were associated with low CAD risk	+	[47]
		High HSP70 levels were associated with increased risk of cardiac death		[48]
Inflammation	Interleukin (IL) such as IL-6 and IL-18	Higher IL-6 levels were associated with SCD and was an independent predictor of sudden death	+++	[51]
		High levels of IL-6 were associated with increased burden of atherosclerosis and higher risk of major adverse cardiac events (MACE) risk		[52]
		Higher IL-6 levels were associated with atherosclerotic cardiovascular disease (ASCVD) events, heart failure (HF) and mortality		[53]
				[55]
		Higher levels of IL-18 and IL-12 were associated with increased risk of cardiovascular events		
	C-reactive protein (CRP)	High CRP levels were associated with greater mortality and risk of cardiovascular disease	++	[60]
		CRP levels were not significantly associated with sudden death and SCD risk		[51, 61]
	Lipoprotein-associated phospholipase A2 (Lp-PLA2)	Higher Lp-PLA2 levels were associated with increased risk of coronary heart disease and was an independent predictor of CHD events	+	[63, 64]
	Myeloperoxidase (MPO)	MPO levels were associated with the incidence and severity of CAD	+	[66]
	Urokinase-type plasminogen activator receptor (uPAR)	High suPAR levels were associated with increased risk of CVD	++	[68, 69]
	Matrix metalloproteinases (MMP)	Higher levels of MMP-1, − 2, − 8 and − 9 were associated with acute coronary syndromes and CAD	+	[74, 75]
	Pentraxin-3 (PTX3)	PTX3 was associated with higher risk of mortality in patients with chronic heart failure	+	[72]
Neurohormonal regulation	Renin and aldosterone	Higher plasma renin and aldosterone levels were associated with increased risk of cardiovascular mortality and adverse outcome in ST-elevation myocardial infarction (STEMI)	+++	[77–79]
	Adrenomedullin (ADM)	High ADM levels were associated with heart failure	++	[81, 82]
		Mid-regional pro–atrial natriuretic peptide (MR-proANP) demonstrated diagnostic and prognostic utility in patients with acute heart failure (AHF)		[80, 84]
	Copeptin	High copeptin levels were associated with increased mortality, readmissions, and emergency department visits in patients with acute heart failure as well as excess mortality in patients with chronic HF	+	[86, 87]
Hemodynamic properties	Natriuretic peptides (NP), i.e. (B-type natriuretic peptide) BNP or (N-terminal pro B-type natriuretic peptide) NT-proBNP	Higher NT-proBNP levels were associated with increased risk of SCD	+++	[61, 89]
		High BNP levels were an independent predictor of sudden death in patient with chronic heart failure		[90]
		High BNP levels were associated with higher risk of death/mortality in patients with acute myocardial infarction		[91]
Myocardial stress, necrosis, fibrosis and tissue regeneration	Cardiac troponins (cTn)	High levels of cTn were associated with the risk of death from cardiovascular causes, myocardial infarction, stroke or heart failure	+++	[92–96]
		High levels of cTn were associated with the severity and progression of chronic heart failure		[97]
	Osteopontin	High osteopontin levels were associated with left ventricular dysfunction and reduced levels were correlated with good response to heart failure therapies	+	[98]
	ST2 receptor	High ST2 levels were associated with cardiovascular mortality in chronic heart failure patients	+	[99]
	Growth differentiator 15 (GDF-15)	High GDF-15 levels were associated with risk of developing CVD and mortality	+	[100]

^a^tab

### Other molecular biomarkers

Apart from genes and proteins, metabolites and other small molecules have also been used as molecular biomarkers for SCD. One good example is reported by Jouven et al., who discovered that non-esterified free fatty acids (NEFAs) could be an independent risk factor for SCD [101]. Meanwhile, elevated levels of trans-18:2 fatty acids were associated with higher risk for SCD in an elderly cohort, whereas higher trans-18:1 with lower risk [102]. F2 isoprostanes are prostaglandin compounds that have shown potential as in vivo markers of oxidant injury in cardiovascular pathologies such as atherosclerosis and acute coronary syndrome (ACS) [103, 104]. Asymmetric Dimethylarginine (ADMA) is an endogenous inhibitor of nitric oxide (NO) production and is significantly associated with risk factors for CVD; showing an independent, strong prognostic value for mortality and future cardiovascular events [105].

### Challenges and future outlook

Development of diagnostics for early detection faces immense challenges. For example, although many candidate biomarkers for SCD have been discovered, so far, early biomarkers remain scarce for the relatively concealed group of high-risk individuals who are asymptomatic and this warrants attention. Besides, SCD has very complex pathophysiology and etiology. Therefore, every candidate biomarker needs to be evaluated in larger cohorts, so that SCD risks can be predicted down to specific clinical sub-groups [1]. Additionally, most cohort studies have used baseline samples that may be irrelevant to events that occurred years later. Hence, repeat measurements throughout the follow-up period are necessary. For omic-scale studies, extensive statistics assessment is necessary. Even so, mere statistical correlations do not automatically imply clinical usefulness. Therefore, candidates obtained from omic studies should be extensively verified with respect to disease pathophysiology and causality. It is also noteworthy that GWAS results may only explain a small fraction of risks and are often inconsistent. As for proteomics, the analysis of biofluids is still plagued by the high complexity and wide dynamic range of protein concentrations in these sample types. Consequently, depletion, enrichment or fractionation techniques are needed to increase the detection of proteins at low abundances. Despite these hurdles, multimarker panels are increasinlgly applied to provide better discrimination of risks of mortality associated with CAD. Beside the afore-mentioned gene panel that targets 174 expertly-selected genes, it was also demonstrated that the combination of plasma levels of multimarkers such as hs-CRP, HSP70, and fibrin degradation products (FDPs) as a biomarker risk score (BRS) can reliably predict CVD events with elevated levels of all three biomarkers [106].

## Conclusion

SCD is a fatal disease that has a very complex etiology. Although a number of risk factors and biomarkers have been used for diagnostics, prognostics and risk stratification for SCD, these biomarkers need to be further evaluated with larger and better-defined cohorts. With omic technologies, the discovery process for biomarkers can be accelerated considerably, especially by using the multi-omics strategy that combines genomics, transcriptomics, proteomics and metabolomics [107]. In addition, since SCD manifests complex phenotypes and pathophysiology, the multimarker panel strategy, with the follow-up in other biophysical tests can be a good combination.

## Abbreviations

ASCVD: Atherosclerotic Cardiovascular Diseases
AV: Atrioventricular
BRS: Biomarker Risk Score
CAD: Coronary Artery Disease
CCTA: Coronary Computed Tomography Angiography
CHD: Coronary Heart Disease
CRP: C-Reactive Protein
CVD: Cardiovascular Disease
ECC: Excitation-Contraction Coupling
ECG: Electrocardiography
FDR: False Discovery Rate
GWAS: Genome-Wide Association Study
HF: Heart Failure
ICC: Inherited Cardiac Condition
ICD: Implantable Cardioverter Defibrillator
LVEF: Left Ventrivle Ejection Fraction
MACE: Major Adverse Cardiac Event
MI: Myocardial Infaction
RAAS: Renin-Angiotensin Aldosterone System
ROS: Reactive Oxygen Species
SA: Sinoatrial
SCD: Sudden Cardiac Death
